# Priapism in sickle cell disease: Associations between *NOS3* and *EDN1* genetic polymorphisms and laboratory biomarkers

**DOI:** 10.1371/journal.pone.0246067

**Published:** 2021-02-04

**Authors:** Camylla Vilas Boas Figueiredo, Rayra Pereira Santiago, Caroline Conceição da Guarda, Rodrigo Mota Oliveira, Luciana Magalhães Fiuza, Sètondji Cocou Modeste Alexandre Yahouédéhou, Suéllen Pinheiro Carvalho, Joelma Santana dos Santos Neres, Antonio Mateus de Jesus Oliveira, Cleverson Alves Fonseca, Valma Maria Lopes Nascimento, Isa Menezes Lyra, Milena Magalhães Aleluia, Marilda Souza Goncalves

**Affiliations:** 1 Instituto Gonçalo Moniz/Fundação Oswaldo Cruz, Salvador, Bahia, Brasil; 2 Universidade Federal da Bahia, Salvador, Bahia, Brasil; 3 Fundação de Hematologia e Hemoterapia da Bahia (HEMOBA), Salvador, Bahia, Brasil; 4 Complexo Hospitalar Universitário Professor Edgard Santos, Salvador, Bahia, Brasil; 5 Universidade Estadual de Santa Cruz, Ilhéus, Bahia, Brasil; University of Alabama at Birmingham, UNITED STATES

## Abstract

Priapism is a urologic emergency characterized by an uncontrolled, persistent and painful erection in the absence of sexual stimulation, which can lead to penile fibrosis and impotence. It is highly frequent in sickle cell disease (SCD) associated with hemolytic episodes. Our aim was to investigate molecules that may participate in the regulation of vascular tone. Eighty eight individuals with SCD were included, of whom thirty-seven reported a history of priapism. Priapism was found to be associated with alterations in laboratory biomarkers, as well as lower levels of HbF. Patients with sickle cell anemia using hydroxyurea and those who received blood products seemed to be less affected by priapism. Multivariate analysis suggested that low HbF and NOm were independently associated with priapism. The frequency of polymorphisms in genes *NOS3* and *EDN1* was not statistically significant between the studied groups, and the presence of the variant allele was not associated with alterations in NOm and ET-1 levels in patients with SCD. The presence of the variant allele in the polymorphisms investigated did not reveal any influence on the occurrence priapism. Future studies involving larger samples, as well as investigations including patients in priapism crisis, could contribute to an enhanced understanding of the development of priapism in SCD.

## Introduction

Sickle cell disease (SCD) is a genetic disorder referring to a group of hemoglobinopathies characterized by the presence of hemoglobin S (HbS) in homozygosis, known as sickle cell anemia (SCA), or by the presence of HbS and hemoglobin C (HbC) in heterozygosis, also termed SC hemoglobinopathy (HbSC) [1].

Clinical manifestations are heterogeneous in SCD and include vaso-occlusive-related events, such as acute chest syndrome and osteonecrosis, as well as hemolysis-related complications, e.g. pulmonary hypertension, leg ulcers, stroke and priapism [2]. Priapism is a clinical manifestation of SCD defined as a prolonged, persistent and often painful penile erection (usually lasting longer than four hours) that is not associated with sexual stimulation [3]. If improperly diagnosed, this clinical condition can lead to penile fibrosis, erectile dysfunction and impotence [4, 5].

Nitric oxide (NO), the main molecule involved in the physiology of erection, plays a critical role in the pathophysiology of priapism [6]. In SCD, ongoing hemolysis induces the release of arginase and free hemoglobin (Hb), contributing to decreased NO bioavailability. Decreased plasma levels of NO negatively regulate the expression of phosphodiesterase type 5 (PDE-5), an intracellular enzyme responsible for cyclic guanosine monophosphate (cGMP) hydrolysis. The sustained production of cGMP causes an unwanted erection, the hallmark of priapism [2, 3, 6, 7].

NO is produced in the vascular milieu through the enzymatic activity of endothelial nitric oxide synthase (eNOS). Polymorphisms in the gene encoding eNOS can modulate plasmatic levels of NO. One of the most clinically relevant mutations is the replacement of the thymine nucleotide by cytosine at the -786 (-786 T/C) position (rs2070744) in the promoter region of the *NOS3* gene. The *NOS3* (rs2070744) (T/C) polymorphism results in reduced eNOS expression and has been clinically associated with cardiovascular events, such as angina and myocardial infarction [8, 9].

In contrast to the vasodilatory effect of NO, endothelin-1 (ET-1), produced by endothelial cells, exhibits prominent vasoconstrictive activity [10]. Polymorphisms in the gene encoding ET-1 (*EDN1*) have been linked to abnormal increases in the concentrations of this peptide. The replacement of a guanine nucleotide with thymine (G/T) in the 5665 region of this gene results in single nucleotide polymorphism *EDN1* (rs5370) of notable clinical relevance. Elevated levels of ET-1 contribute to endothelial dysfunction and have been associated with pulmonary hypertension, cardiovascular disease and pre-eclampsia [11–14].

The chronic hemolytic feature of SCD is endothelial dysfunction, which is also the basis of priapism [7]. In this context, several laboratory biomarkers are used to evaluate SCD severity. Fetal hemoglobin (HbF), the most important modulator of the clinical and hematologic features of SCD, is unable to form the HbS polymer and reduces mean corpuscular HbS concentrations, thereby inhibiting episodes of erythrocyte sickling. Hence, drugs capable of increasing HbF levels, such as hydroxyurea (HU), are commonly used in the treatment of SCD [15, 16].

In an effort to contribute to a better understanding of the mechanisms involved in the establishment of priapism events, the present study aimed to investigate associations between polymorphisms in *NOS3* and *EDN1* genes, serum levels of NO and ET-1 and laboratory biomarkers in individuals with SCD with a history of priapism.

## Methods

### Subjects

All patients were recruited at the Bahia Hemotherapy and Hematology Foundation (HEMOBA), from October 2016 to September 2017. We included all male patients who underwent routine laboratory tests and clinical examination at the pediatric outpatient clinic of the HEMOBA. A group of 37 male patients, aged 12.7 ± 5.5 years, with SCD (age range 14 [IQR: 9.5–16] years, 31 SCA and 6 HbSC) who reported episodes of priapism (priapism+) were investigated in a case-control study. In addition, a control group consisting of 51 male patients, aged 14.6 ± 3.5 years, with SCD (age range 15 [12–17] 36 SCA and 15 HbSC) who did not develop priapism events (priapism-) were also included. All patients were age-matched.

Data on priapism events were obtained through the application of a standard questionnaire and the review of patient medical records. Priapism was clinically defined as persistent and painful penile erection lasting at least two hours, occurring once or multiple times a day, requiring medication, as well as severe episodes requiring hospitalization. Of the 37 priapism+ patients involved in the study, three underwent priapism-related surgery based on Chester Winter procedure. Last priapism episode was at least three months prior to blood sample.

None of the patients who participated in the study presented any acute SCD events, or infections in the three months prior to inclusion, characterizing a steady-state of disease. The presence of infectious diseases and hemoglobin variants other than HbS or HbC were used as exclusion criteria.

This study was approved by the São Rafael Hospital institutional research board (protocol number: CAAE 52280015.1.0000.0048), and all individuals and their legal guardians signed a term of informed consent. All study protocols were performed in accordance with the Helsinki Declaration of 1975 and subsequent revisions.

### Biochemical and hematological analysis

Biochemical analysis was performed using an A25 Clinical Autoanalyzer (Biosystems S.A, Barcelona, Catalunya, Spain) in accordance with the manufacturer's instructions. Hematological parameters were evaluated using a Beckman Coulter LH 780 Hematology Analyzer (Beckman Coulter, Brea, California, USA). Hemoglobin profiling was performed by High Performance Liquid Chromatography (HPLC) using a Variant II system (Bio-Rad, California, USA). Reticulocytes were counted after staining supravitally with brilliant cresyl blue dye.

### Genetic analysis

Genomic DNA was extracted from white blood cells using a QIAamp DNA Blood Mini Kit (Qiagen, California, San Francisco, USA) in accordance with the manufacturer's instructions. Polymorphisms *NOS3* (rs2070744) and *EDN1* (rs5370) were investigated by polymerase chain reaction (PCR) via a combination of specific primers and PCR product digestion using the restriction fragment length polymorphism technique (RFLP), as previously described [17, 18] Allele-specific PCR was used to investigate the -α^3.7Kb^-thal as previously described [19].

### ET-1 assay

ET-1 quantification was performed in serum samples by enzyme linked immunosorbent assay (ELISA) following the manufacturer's instructions (R&D Systems, Minneapolis, USA).

### NO metabolites assay

NO metabolite (NOm) levels were evaluated by the Griess reaction using colorimetry, and compared to a sodium nitrate (NaNO_3_) standard-curve, as previously described [20].

### Statistical analysis

Statistical analyses were conducted using software programs Statistical Package for the Social Sciences (SPSS) version 21.0 (IBM Software, New York, USA) and GraphPad Prism version 6.0 (Graphpad Software, California, USA), which was also used for graph assembly. *p* values < 0.05 were considered significant. The Kolmogorov-Smirnov test was used to analyze quantitative variables with normal distributions. The independent t-test and Mann–Whitney *U* test were used to compare two numerical variables between groups in accordance with the distribution of each variable. Fisher’s two-tailed exact test was used to evaluate associations between qualitative or categorical variables. Multivariate binary logistic regression analysis was performed to evaluate possible associations between gene polymorphisms and laboratory biomarkers with regard to the outcome of interest, priapism.

## Results

### Laboratory parameters

Investigation of laboratory parameters in SCD priapism+ and priapism- revealed that priapism+ patients exhibited increased NOm, alkaline phosphatase (ALP), red blood cell distribution width (RDW) and mean corpuscular hemoglobin concentration (MCHC) levels, as well as lymphocyte counts. In addition, SCD priapism+ patients presented lower endothelin levels (S1 Table). We further decided to investigate each SCD genotype.

**Fig 1:**
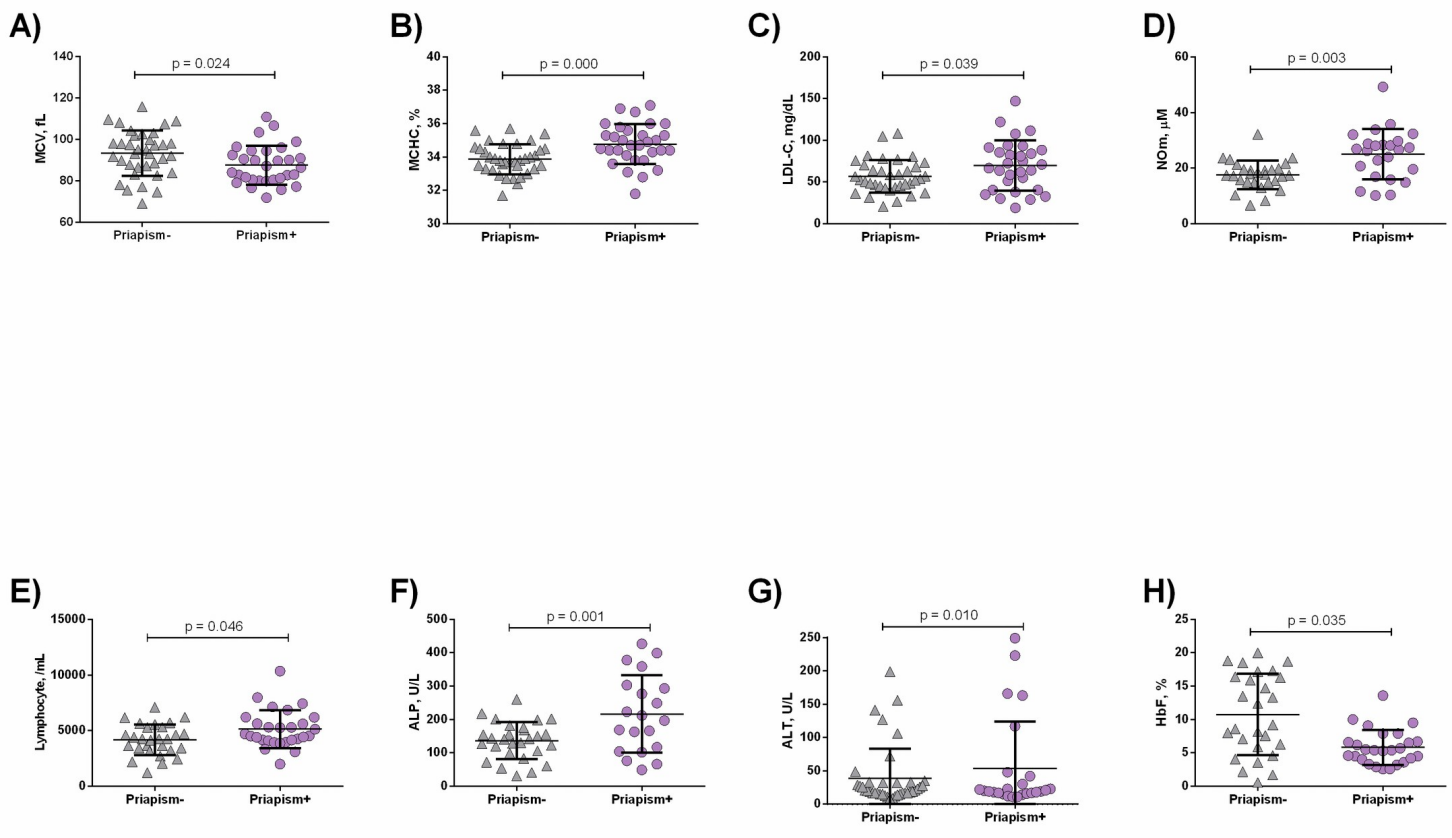
Laboratory parameters in SCA patients priapism- and priapism+. Priapism+ presented decreased A) VCM levels; B) increased MCHC; C) LDL-C; D) NOm levels; E) Lymphocyte counts; F) ALP; G) ALT levels, in addition to decreased H) HbF levels. Significant p values were obtained with Independent t test as well as Mann Whitney *U* test.

SCA priapism+ patients presented lower mean corpuscular volume (MCV) (p = 0.024, Fig 1A), in addition to increased MCHC (p = 0.000, Fig 1B), low-density lipoprotein cholesterol (LDL-C) (p = 0.039, Fig 1C) and NOm (p = 0.003, Fig 1D) levels, as well as higher lymphocyte counts (p = 0.046, Fig 1E) than the priapism- group. SCA priapism+ patients also had higher levels of ALP compared to the priapism- group (p = 0.001, Fig 1F) in addition to decreased alanine aminotransferase (ALT) levels (p = 0.010, Fig 1G). HbF levels were decreased in priapism+ compared to priapism+ (p = 0.035, Fig 1H).

**Fig 2:**
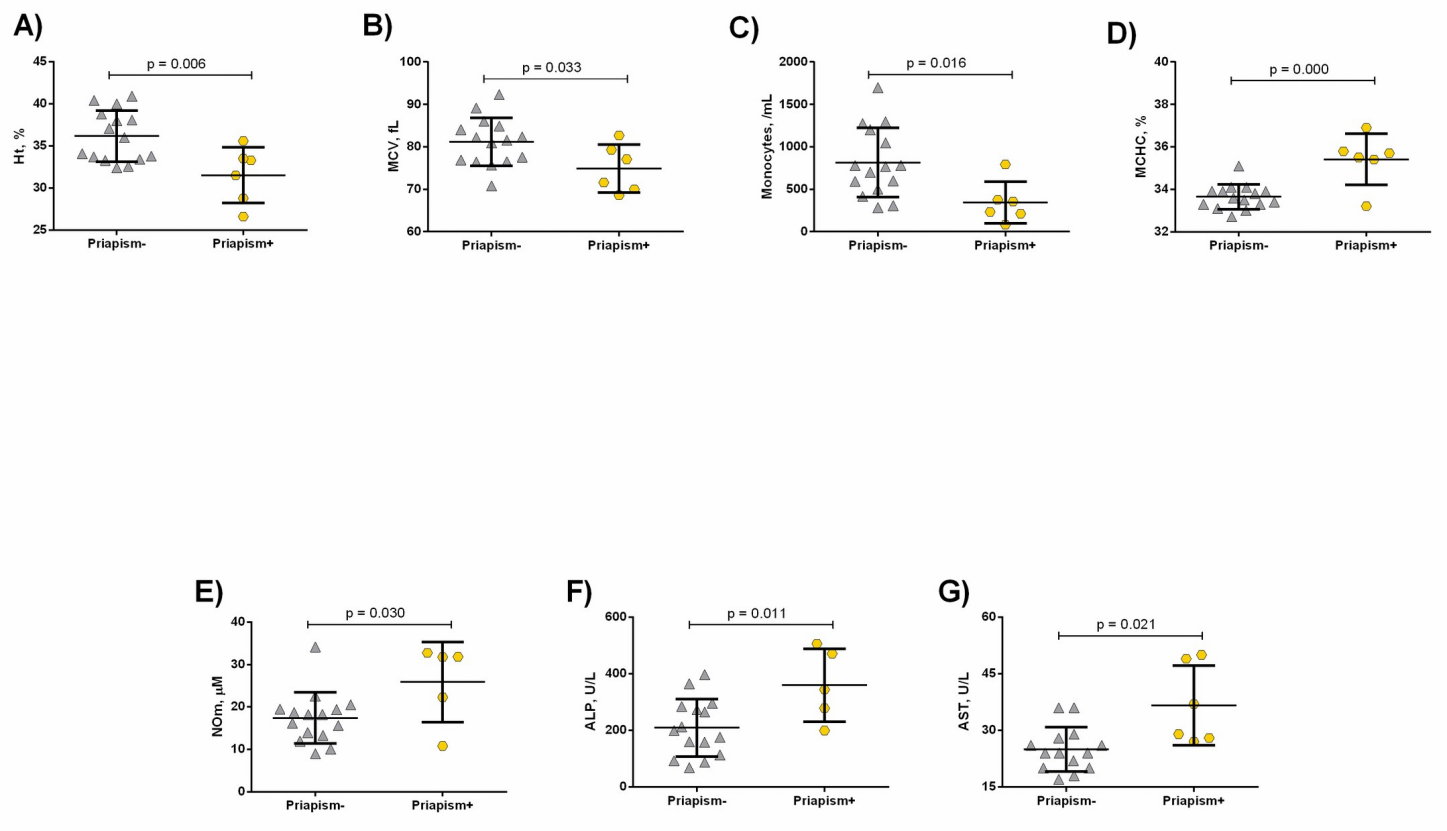
Laboratory parameters in HbSC patients priapism- and priapism+. Priapism+ presented decreased A) Hematocrit; B) MCV levels, in addition to decreased C) monocyte counts. Moreover, priapism+ exhibited increased D) MCHC; E) NOm; F) ALP; G) AST levels. Significant p values were obtained with Independent t test as well as Mann Whitney *U* test.

HbSC priapism+ patients exhibited decreased hematocrit (p = 0.006, Fig 2A), MCV (p = 0.033, Fig 2B) and monocyte counts (p = 0.016, Fig 2C), as well as increased MCHC (p = 0.000, Fig 2D) and NOm levels (p = 0.030, Fig 2E) compared to priapism-. In addition, higher levels of ALP (p = 0.011, Fig 2F) and aspartate aminotransferase (AST) (p = 0.021, Fig 2G) were detected in the HbSC priapism+ patients compared to the priapism- group.

All laboratory parameters investigated are described in S1–S3 Tables. Median and interquartile range of laboratory parameters are described in S2 Table whereas mean and standard deviation are described in S3 Table.

### Association between priapism and HU therapy or history of blood transfusion

Clinical manifestations of each genotype are described in Table 1. SCA patients priapism+ had more episodes of stroke, acute pain crises, acute chest syndrome, vaso-occlusive episodes and received more blood transfusions than priapism- patients. α^3.7kb^ thalassemia was more prevalent in priapism- patients, although no statistical difference was found. Moreover, HbSC patients priapism+ presented more prevalent vaso-occlusive episodes and acute pain crises than patients priapism-, although no statistical difference was found.

**Table 1:** Clinical characterization of SCA and HbSC patients with and without previous history of priapism.

Clinical Characteristic	SCA			HbSC		
	Priapism + (n = 31)	Priapism–(n = 36)	*P*	Priapism + (n = 6)	Priapism–(n = 15)	*p*
**Stroke**	3	1	0.329	0	1	1.000
**Acute pain crises**	23	22	0.304	6	14	1.000
**Acute chest syndrome**	10	8	0.075	0	4	1.000
**Vaso-occlusive episode**	18	12	0.052	4	1	0.011
**α**^**3.7kb**^ thalassemia genotype*	4	9	0.750	0	2	1.000
**Blood transfusion**	14	7	**0.035**	1	0	0.286
**HU therapy**	6	18	**0.011**	0	2	1.000

*Frequencies are shown for homozygous and heterozygous for the α^3.7kb^ deletion. P-value obtained with Fisher’s two-tailed exact test.

**Fig 3:**
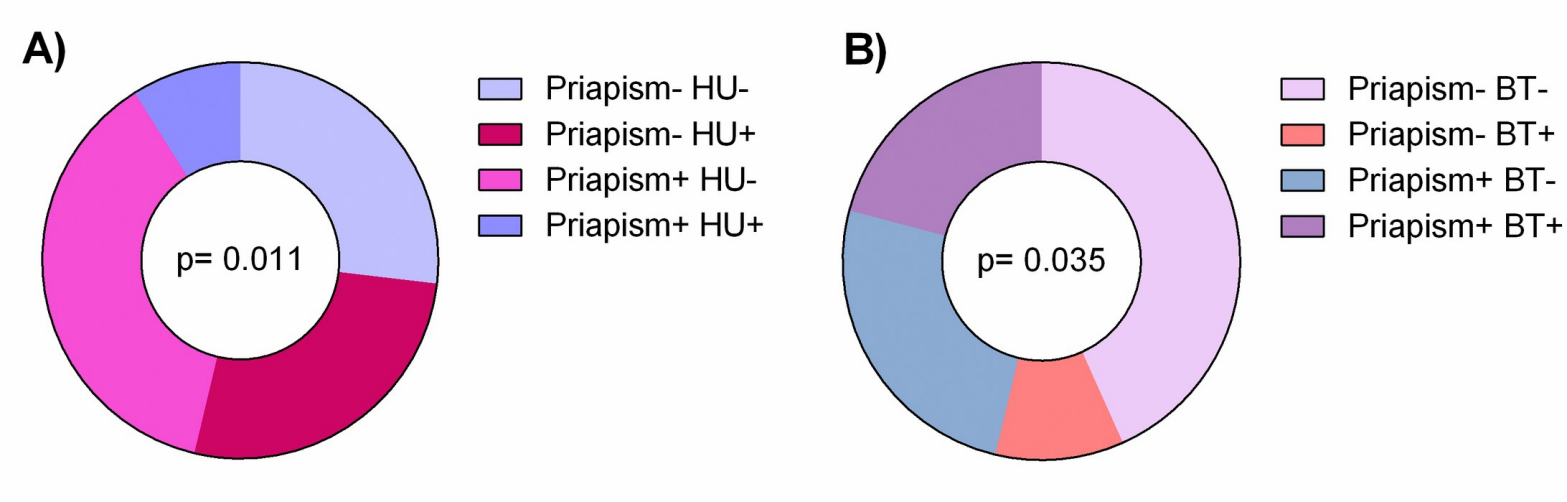
Treatment with HU or BT was associated with frequency of priapism in SCA patients. Significant p values were obtained with Fisher’s two-tailed exact test.

Our results demonstrated that patients with SCA treated pharmacologically with HU were less affected by priapism events (p = 0.011). In addition, blood transfusions was more prevalent among priapism+ (p = 0.035). This association may be explained because blood transfusion is a therapeutic approach for priapism; moreover three patients were in exchange transfusion due to previous stroke (Fig 3).

### Polymorphism frequencies

The genotypic frequencies of the *EDN1* 5665 G/T and *NOS3*–786 T/C polymorphisms in both SCA and HbSC priapism+ and priapism- groups are described in Table 2. The analysis of *EDN1* 5665 G/T revealed that the homozygous wild type (GG) was the most frequent, while the homozygous mutant (TT) presented the lowest frequency in all groups. Moreover, no significant differences were seen between the genotypic frequencies of individuals with or without a previous history of priapism, for both the SCA and HbSC groups.

**Table 2:** Frequencies of *EDN1* 5665 G/T and *NOS3*−786 T/C gene polymorphisms among SCA and HbSC individuals with or without a previous history of priapism.

Polymorphism	Genotype	SCA individuals (%)			HbSC individuals (%)		
		Priapism+	Priapism-	*p*	Priapism+	Priapism-	*p*
***EDN1* 5665 G/T**	GG	71 (22/31)	69.4 (25/36)	1.00	83.3 (5/6)	80 (12/15)	1.00
	GT	22.6 (7/31)	27.8 (10/36)	0.779	16.7 (1/6)	20 (3/15)	1.00
	TT	6.4 (2/31)	2.8 (1/36)	1.00	0 (0/6)	0 (0/15)	ND
***NOS3–*786 T/C**	TT	58 (18/31)	55.6 (20/36)	1.00	50 (3/6)	66.7 (10/15)	0.675
	TC	42 (13/31)	44.4 (16/36)	1.00	50 (3/6)	33.3 (5/15)	0.631
	CC	0 (0/31)	0 (0/36)	ND	0 (0/6)	0 (0/15)	ND

ND: not determined. Both polymorphisms were found to be in Hardy–Weinberg equilibrium. p values obtained with Fisher’s two-tailed exact test.

Regarding the *NOS3*–786 T/C polymorphism, the homozygous wild type (TT) was also found to be the most frequent, while the homozygous mutant genotype was not identified in our cohort. Again, no significant differences in genotypic frequencies were detected between priapism+ and priapism- patients in either of the SCA or HbSC groups.

### Associations between ET-1 and NOm levels in the *EDN1* 5665 G/T and *NOS3*–786 T/C polymorphisms

**Fig 4:**
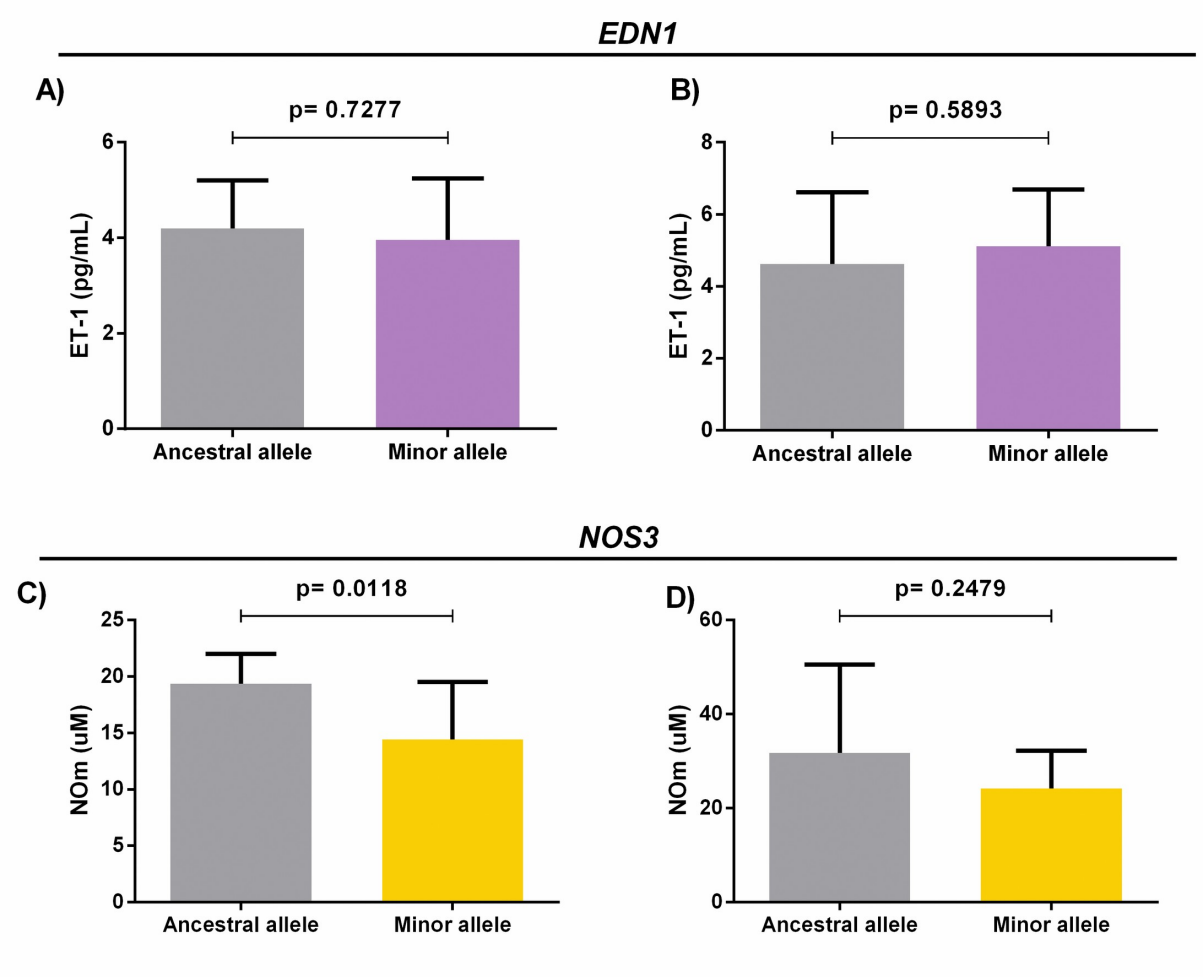
A) Analysis of ET-1 levels and the presence of ancestral and minor alleles in the *EDN1* 5665 G/T gene polymorphism among SCA patients with priapism. B) Analysis of ET-1 levels and the presence of ancestral and minor alleles in the *EDN1* 5665 G/T gene polymorphism in SCA patients without priapism. (Independent t-test). C) Analysis of NOm levels and the presence of ancestral and minor alleles in the *NOS3*–786 T/C gene polymorphism among SCA patients with priapism. D) Analysis of NOm levels and the presence of ancestral and minor alleles in the *NOS3*–786 T/C gene polymorphism in SCA patients without priapism (Independent t-test).

We found no significant differences between ET-1 levels and the ancestral and minor alleles of *EDN1* 5665 in the SCA priapism+ and priapism- groups (Fig 4A and 4B). However, our results showed statistically significant differences between NOm levels in the ancestral and minor alleles of *NOS3*–786 in the SCA priapism- group (p = 0.0118) (Fig 4C); no similar association was observed among priapism+ patients (Fig 4D).

### Multivariate analysis of associations between gene polymorphisms and laboratory biomarkers among SCA priapism+ patients

Our multivariate analysis models were designed to investigate any associations between genetic polymorphisms and laboratory parameters, using priapism as dependent variable. Two multivariate analysis models were found to be statistically significant (p = 0.015 and p = 0.000 respectively, Table 3). For Model 1, R^2^ was 0.27, for Model 2 R^2^ was 0.25. Our models have shown that HbF and NOm levels were independently associated with priapism (Table 3). No significant associations were found with respect to the other independent variables investigated.

**Table 3:** Multivariate analysis of associations between gene polymorphisms and laboratory biomarkers in SCA and HbSC patients with a previous history of priapism.

Variables	B	SE	Wald	95% IC	*p*	*p* of model
Model 1						
HbF, %	1.829	0.897	4.158	1.074–36.13	**0.041**	**0.015**
*EDN1* 5665 G/T	-0.769	0.759	1.025	0.105–2.053	0.311	
ET-1, pg/mL	-0.692	0.671	1.064	0.134–1.865	0.302	
*NOS3–*786 T/C	0.449	0.680	0.437	0.413–5.941	0.509	
NOm, μM	-0.998	0.692	2.081	0.095–1.430	0.149	
Model 2						
Blood transfusion +	1.560	0.875	3.179	0.857–26.448	0.075	
HU +	-0.837	0.757	1.223	0.098–1.908	0.269	
HbF, %	-0.035	0.072	0.230	0.839–1.112	0.632	**0.000**
ET-1, pg/mL	-1.061	0.682	2.421	0.091–1.317	0.120	
NOm, μM	-1.330	0.628	4.484	0.077–0.906	**0.034**	

B: coefficient; SE: standard error. Significant p values are shown in bold.

## Discussion

Priapism, defined as a prolonged and painful penile erection not associated with sexual desire, is considered an urgent urological condition, since late diagnosis can trigger a series of complications, including penile fibrosis and erectile dysfunction [7]. While this condition has been associated with several hemolytic anemias, such as thalassemia, hereditary spherocytosis and paroxysmal nocturnal hemoglobinuria (PNH), the highest incidence is observed in patients with SCD [4, 15]. An international study, involving five reference SCD treatment centers in the United Kingdom and Nigeria, interviewed 130 men with SCD and found that 46 (35%) experienced at least one priapism event during their lifetime, while 10 (21%) reported having developed erectile dysfunction [21].

In the context of a hemolytic phenotype, priapism events are associated with changes in laboratory biomarkers related to intravascular hemolysis. Studies have shown that SCA patients with a prior history of priapism presented increased levels of lactate dehydrogenase (LDH), AST, bilirubin and reticulocytes compared to unaffected patients [4, 7, 22, 23]. However, data in the literature indicates this is not always the case. Madu et al. [24] investigated the medical records of 126 patients with SCA seen at a university medical center in Nigeria, and found that 21.4% of the study group presented priapism. Among the laboratory markers investigated, only leukocyte counts were statistically significant between patients with priapism and controls. No significant differences were observed among classical markers of hemolysis, such as hemoglobin (Hb) concentration, bilirubin (direct and indirect) and AST. Another study involving 415 patients with SCA recruited from health centers in the United States and the United Kingdom used principal component analysis to derive a hemolytic component value considering reticulocyte count, serum levels of LDH, AST, and total bilirubin. These authors also found no associations between markers of hemolysis and the occurrence of priapism [25].

In the patients studied herein, no direct associations were found between the hemolysis parameters evaluated and a prior history of priapism. Only AST level was found to be statistically higher in HbSC individuals who had priapism compared to controls. However, changes in hematimetric index values, such as increased MCHC and decreased MCV, as well as higher levels of ALP, were observed in both SCA and HbSC patients who had priapism compared to controls. We were unable to identify any published studies in the literature that described laboratory alterations associated with priapism in SCD, although significant evidence of intravascular hemolysis, sickle cell vasculopathy and priapism have been suggested [7]. LDL-C levels were found to be increased in SCA priapism+ individuals, which may be associated with previously reported altered lipid metabolism in SCA [26–28] in addition to the potential pro-inflammatory role of LDL-C in the intravascular microenvironment [29].

HbF is an important modulator of SCD phenotype. Since this type of hemoglobin does not participate in HbS polymer formation, higher levels of HbF reduce HbS polymerization as well as mean corpuscular volume of HbS, thereby impairing the erythrocyte sickling process. Increased HbF concentrations also diminish the occurrence of various related clinical manifestations, including acute chest syndrome, leg ulcers and cholelithiasis, and have been associated with prolonged survival in individuals with SCD [30–32]. While the influence of HbF on priapism is not well-established in the literature, some studies have shown no statistical differences between HbF levels in patients with SCD who had priapism in comparison to control groups [7, 33].

Our results demonstrate that patients with SCA who had prior episodes of priapism presented lower HbF concentrations compared to controls. Furthermore, multivariate analysis of several of the investigated hemolytic and biochemical parameters revealed that HbF was independently associated with a previous history of priapism.

Pharmacological treatment with HU and the use of blood products are therapies capable of modifying the clinical course of individuals with SCD. HU exerts several beneficial effects, including increased expression of HbF and decreased leukocyte counts. Clinically, HU has been shown to reduce the incidence of vaso-occlusive crises, hospitalizations, and SCD-related mortality [34]. For acute priapism episodes, different pharmacological strategies may be useful, such as vasoactive agents, including both α and β-adrenergic agonists in addition to management with hydration and analgesia [35]. HU is thought to act as a strategy for secondary prevention of priapism episodes, although the efficiency long-term therapy is unclear [35]. Here we noted that subjects with SCA pharmacologically treated with HU were less affected by previous episodes of priapism.

The use of blood products has been associated with enhanced microvascular flow rate due to decreased numbers of circulating sickle erythrocytes, as well as decreased inflammation and endothelial injury [1, 34]. Studies have shown that the prophylactic use of blood products prevents the occurrence of stroke by 90% in children with increased cerebral blood velocity, as determined by transcranial Doppler. The beneficial effect of prophylactic transfusion is associated with improved blood oxygenation capacity and reduced HbS concentrations, thereby implying a reduction in the frequency of hemolytic episodes [1, 36]. Our results show that subjects with SCA priapism+ presented increased frequency of other clinical manifestations as well as blood transfusion therapy. In a follow-up study, ten patients were followed for twenty four years and blood exchange transfusion seemed to be efficient and safe for priapism treatment. The same study also found that after blood exchange transfusion hemoglobin levels were about 10 g/dL as well as HbS levels were below 30% [37]. In our casuistic, patients received blood transfusion after priapism episode (as a therapy and preventive approach) and three patients were under exchange transfusion due to previous stroke.

The mechanism underlying priapism events is closely related to the bioavailability of NO [15]. Our data shows that levels of NO metabolites (NOm) in SCA and HbSC patients with a previous history of priapism were higher compared to controls. This result stands in contrast to data in the literature indicating that decreased bioavailability of NO was directly associated with the priapism event [7]. Moreover, NOm levels were shown to be independently associated with priapism in multivariate analyses. NO pathway in intravascular spaces plays a complex role. Evidence from transgenic mice has suggested that NO imbalance may contribute for priapism onset [38]. Our results have shown that patients priapism+ presented increased NOm levels. We believe that this may be justified by multiple reasons. First, patients priapism+ may produce NOm in a compensatory fashion, due to increased intravascular hemolysis. Second, the Griess reaction detects both nitrite and nitrate in patients’ sample; thus, higher NOm levels may reflect increased nitrosative stress [20, 39]. Third, although the NO-deficiency is a hallmark of SCD physiopathology, many authors have found that SCD subjects presented higher whole blood, red blood cells and plasma levels of nitrite when compared to control subjects [40, 41]. In addition, the individuals studied herein were not evaluated in the context of priapism crisis. The precise underlying mechanism involved in altered NO signaling pathway in the penis leading to priapism in SCD patients remains unclear.

Since priapism is an event related to endothelial dysfunction [7], it is therefore important to investigate molecular parameters with potential involvement in order to achieve a better understanding of the process underlying this clinical manifestation. Synthesized by endothelial cells, endothelin-1 (ET-1) is a peptide that exerts potent vasoconstrictive activity [10]. Its release is mediated by inflammatory cytokines, as well as by endothelial injury or activation. Thus, due to the presence of a chronic inflammatory state and endothelial dysfunction, increased levels of ET-1 are an expected finding in SCD [42]. Higher levels of ET-1 were found in SCA patients in acute pain and vaso-occlusive crises, as well as in bone pain crisis, compared to controls [43–45]. While the literature contains no studies attempting to associate ET-1 levels with the occurrence of priapism in SCD, our results showed no statistical differences in ET-1 concentrations between SCA and HbSC patients with a previous history of priapism compared to controls.

Priapism has been associated with some genetic markers in SCD, including Klotho, factor XIII, integrin α_V_ and transforming growth factor β receptor-3 [46], which may modulate the endothelial vascular biology. The *NOS3* (rs2070744) polymorphism, which results from the nucleotide substitution of thymine by cytosine (T/C) at position -786, has been associated with a reduction in the expression of eNOS, and is clinically linked to vascular disorders such as myocardial infarction, angina, atherothrombosis, erectile dysfunction, stroke and kidney disease [8, 9, 47–50]. Studies demonstrating the influence of this polymorphism on SCD modulation differ among the populations investigated. Sharan et al. [51] reported that the presence of this polymorphism is associated with increased acute chest syndrome development in African American women with SCD. Studies involving Brazilian patients with SCD have demonstrated that the inheritance of a variant allele is related to the occurrence of infections in the upper respiratory tract, as well as to a more severe clinical course of disease [52, 53]. Nishank et al. [54] found that the presence of this polymorphism in Indian patients was associated with SCD. In addition, in another study [55], Nishank et al. reported that the presence of this polymorphism in combination with lower NOm levels could influence the late onset of menarche in Indian adolescents with SCD. On the other hand, Thakur et al. [56] investigated 145 African SCA patients and found no statistical differences in the allelic frequency of this polymorphism compared to controls.

Some genetic polymorphisms have been associated with changes in serum ET-1 levels. Studies have shown that higher levels of ET-1 were linked to the presence of the *EDN1* 5665 polymorphism, which leads to the substitution of the guanine nucleotide for thymine [11, 17]. This mutation was also associated with pulmonary dysfunction and severe VOC in Egyptian pediatric patients with SCD [57]. Thakur et al. [56] identified a statistical difference in the frequency of this polymorphism between individuals with SCD and controls. Herein, we observed no statistical differences between serum levels of ET-and genotypic frequency, nor the presence of the variant allele. Due to small n our study failed to find statistical significance between the polymorphisms and history of priapism in SCD patients, although different studies also presented a reduced number of enrolled subjects [16, 37]. Moreover, our results are in agreement with the complex role of NO pathway in regulation of vascular tonus, in addition to the well documented association between intravascular hemolysis and endothelial dysfunction.

## Conclusions

The present study found that a prior history of priapism may be associated with alterations in laboratory biomarkers in SCA and HbSC patients. HbF was shown to be relevant to a prior history of priapism, since lower serum levels were found in individuals who suffered from this condition compared to controls. In addition, patients who used HU also appeared to be less affected by priapism events. Our investigation of polymorphisms in genes *NOS3* and *EDN1* revealed no statistical differences among the individuals studied, and the presence of the variant allele was not associated with alterations in serum levels of ET-1 or NOm in individuals with a previous history of priapism. Future studies involving a larger number of participants and the inclusion of patients undergoing priapism crisis should provide further clarification regarding the role played by these molecules in the development of this clinical manifestation.

## Supporting information

S1 Table Laboratory profiles of SCD individuals with or without a previous history of priapism. (DOCX)

S2 Table Laboratory profiles of SCA and HbSC individuals with or without a previous history of priapism (median and interquartile range). (DOCX)

S3 Table Laboratory profiles of SCA and HbSC individuals with or without a previous history of priapism (mean and standard deviation). (DOCX)
