# Supplementary material for: Priapism in sickle cell disease: Associations between NOS3 and EDN1 genetic polymorphisms and laboratory biomarkers
Source: PLoS One. 2021 Feb 4;16(2):e0246067. doi: 10.1371/journal.pone.0246067 (PMC7861393; doi:10.1371/journal.pone.0246067)
Supplement: S1 Table — (DOCX) [file pone.0246067.s001.docx]

**S1 Table.** Laboratory profiles of SCD individuals with or without a previous history of priapism

|  | **Priapism +**  **(n = 37)** | **Priapism –**  **(n = 51)** | |
| --- | --- | --- | --- |
|  | **Median (IQR)** | **Median (IQR)** | ***p*** |
| **Age, years** | 14 (9.5 – 16) | 15 (12 – 17) | - |
| **RBC, x10^6^/mL** | 2.80 (2.56 – 3.38) | 2.79 (2.53 – 4.17) | 0.606^#^ |
| **Hemoglobin, g/dL** | 8.40 (7.60 – 9.80) | 8.90 (7.90 – 11.40) | 0.211^#^ |
| **Hematocrit, %** | 24.50 (21.75 – 28.75) | 27.00 (22.60 – 33.30) | 0.054^#^ |
| **MCV, fL** | 82.80 (79.15 – 91.70) | 89.20 (81.60 – 97.90) | 0.064 |
| **MCH, pg** | 29.50 (27.40 – 31.35) | 30.10 (27.40 – 32.80) | 0.473 |
| **MCHC, %** | 35.0 (34.20 – 33.75) | 33.80 (33.30 – 34.30) | **0.000** |
| **RDW, %** | 23.50 (19.50 – 27.55) | 21.30 (17.80 – 24.40) | **0.019** |
| **Reticulocyte, %** | 4.80 (3.60 – 6.25) | 4.70 (3.10 – 6.00) | 0.437^#^ |
| **Total bilirubin, mg/dL** | 2.99 (1.39 – 4.30) | 2.85 (1.59 – 3.61) | 0.476^#^ |
| **Direct bilirubin, mg/dL** | 0.39 (0.29 – 0.50) | 0.35 (0.26 – 0.50) | 0.385 |
| **Indirect bilirubin, mg/dL** | 2.46 (1.00 – 3.90) | 2.63 (1.23 – 3.20) | 0.498 |
| **LDH, U/L** | 1037 (848.3 – 1659) | 932 (618 – 1361) | 0.051^#^ |
| **CRP, mg/L** | 4.19 (2.43 – 6.09) | 3.15 (1.95 – 5.95) | 0.420^#^ |
| **HbF, %** | 4.95 (3.35 – 7.60) | 5.05 (1.67 – 13.35) | 0.986^#^ |
| **WBC, /mL** | 11600 (9900 – 12700) | 10900 (7800 – 13700) | 0.291 |
| **Neutrophil, /mL** | 4554 (3489 – 6018) | 5060 (3100 – 7300) | 0.685^#^ |
| **Eosinophil, /mL** | 468 (265 – 862) | 400 (182 – 756) | 0.256^#^ |
| **Lymphocyte, /mL** | 4532 (3947 – 5816) | 3672 (3013 – 4925) | **0.006**^#^ |
| **Monocyte, /mL** | 800 (420 – 1158) | 900 (663 – 1296) | 0.107^#^ |
| **Platelet, /mL** | 418 (322 – 467) | 409 (320 – 489) | 0.410 |
| **Total cholesterol, mg/dL** | 124.0 (100.0 – 146.8) | 112.0 (97.0 – 128.0) | 0.320 |
| **HDL-C, mg/dL** | 34.0 (29.2 – 41.0) | 36.0 (32.0 – 42.0) | 0.293^#^ |
| **LDL-C, mg/dL** | 65.9 (41.7 – 90.6) | 58.8 (44.6 – 74.2) | 0.162 |
| **VLDL-C, mg/dL** | 21.6 (14.6 – 28.0) | 19.2 (14.4 – 22.8) | 0.114 |
| **Triglycerides, mg/dL** | 108.0 (73.0 – 138.0) | 96.0 (72.0 – 114.0) | 0.120 |
| **ALT, U/L** | 19.0 (15.5 – 23.0) | 17.0 (12.0 – 27.5) | 0.484^#^ |
| **AST, U/L** | 48.50 (38.25 – 58.75) | 39.0 (26.0 – 64.0) | 0.097^#^ |
| **ALP, U/L** | 236.0 (141.0 – 383.3) | 145.0 (99.0 – 201.0) | **0.002**^#^ |
| **NOm, µM** | 27.13 (18.97 – 32.13) | 18.06 (14.84 – 20.81) | **0.000**^#^ |
| **ET-1, pg/mL** | 4.04 (3.32 – 4.76) | 4.64 (4.02 – 6.01) | **0.032**^#^ |

RBC: Red blood cells; MCV: mean cell volume; MCH: mean corpuscular hemoglobin; MCHC: mean corpuscular hemoglobin concentration; RDW: red cell distribution; LDH: lactate dehydrogenase; CRP: C-reactive protein; HbF: Fetal hemoglobin; WBC: white blood cell; HDL-C: high-density lipoprotein cholesterol; LDL-C: low-density lipoprotein cholesterol; VLDL-C: very low-density lipoprotein cholesterol; ALT: Alanine aminotransferase; AST: Aspartate aminotransferase; ALP: Alkaline phosphatase; NOm: nitric oxide metabolites; ET-1: endothelin-1. IQR: Interquartile range. Significant p values are shown in bold. p value obtained with Independent t test or ^#^p value obtained with Mann-Whitney *U* test.
