# Supplementary material for: Priapism in sickle cell disease: Associations between NOS3 and EDN1 genetic polymorphisms and laboratory biomarkers
Source: PLoS One. 2021 Feb 4;16(2):e0246067. doi: 10.1371/journal.pone.0246067 (PMC7861393; doi:10.1371/journal.pone.0246067)
Supplement: S2 Table — (DOCX) [file pone.0246067.s002.docx]

**S2 Table.** Laboratory profiles of SCA and HbSC individuals with or without a previous history of priapism (median and interquartile range).

|  | **SCA individuals (n= 67)** | | | **HbSC individuals (n=21)** | | |
| --- | --- | --- | --- | --- | --- | --- |
|  | **Priapism+**  **(n=31)** | **Priapism-**  **(n=36)** |  | **Priapism+**  **(n=6)** | **Priapism-**  **(n=15)** |  |
|  | **Median (IQR)** | **Median (IQR)** | ***P*** | **Median (IQR)** | **Median (IQR)** | ***p*** |
| **Age, years** | 15 (10 – 17) | 14.5 (11 – 17) | - | 9.5 (2.7 – 14.5) | 15.0 (14.0 – 17.0) | - |
| **RBC, x10^6^/mL** | 2.70 (2.48 – 3.06) | 2.63 (2.34 – 2.94) | 0.352^#^ | 4.15 (3.93 – 4.60) | 4.36 (4.17 – 4.78) | 0.253 |
| **Hemoglobin, g/dL** | 8.30 (7.50 – 9.20) | 8.50 (7.50 – 9.07) | 0.940^#^ | 11.20 (10.10 – 12.38) | 11.90 (11.40 – 13.20) | 0.066 |
| **Hematocrit, %** | 23.90 (21.20 – 27.50) | 24.80 (22.30 – 27.65) | 0.532 | 32.40 (28.25 – 34.03) | 36.0 (33.40 – 38.80) | **0.006** |
| **MCV, fL** | 86.40 (80.40 – 94.50) | 93.95 (86.73 – 101.4) | **0.024** | 74.35 (69.60 – 80.15) | 81.60 (76.50 – 84.90) | **0.033** |
| **MCH, pg** | 29.80 (28.20 – 32.90) | 32.05 (29.45 –34.33) | 0.175 | 26.40 (24.88 – 27.83) | 27.40 (25.70 – 28.60) | 0.404 |
| **MCHC, %** | 34.70 (34.10 – 35.60) | 33.80 (33.30 – 34.50) | **0.000** | 35.60 (34.85 – 36.08) | 33.60 (33.30 – 33.90) | **0.000** |
| **RDW, %** | 23.90 (21.30 – 28.60) | 23.15 (20.43 – 25.05) | 0.092 | 19.40 (18.40 – 19.65) | 17.60 (16.0 – 19.80) | 0.237 |
| **Reticulocyte, %** | 5.00 (4.10 – 6.80) | 5.65 (3.62 – 6.55) | 0.895^#^ | 3.05 (2.17 – 4.65) | 3.20 (2.40 – 4.30) | 0.906 |
| **Total bilirubin, mg/dL** | 3.52 (2.23 – 4.38) | 3.23 (2.05 – 3.88) | 0.726 | 1.24 (0.97 – 1.95) | 1.45 (0.89 – 1.92) | 0.863 |
| **Direct bilirubin, mg/dL** | 0.43 (0.30 – 0.54) | 0.38 (0.27 – 0.52) | 0.301 | 0.24 (0.10 – 0.30) | 0.29 (0.23 – 0.42) | 0.151 |
| **Indirect bilirubin, mg/dL** | 2.76 (1.80 – 3.95) | 2.94 (1.54 – 3.38) | 0.810 | 1.05 (0.77 – 1.65) | 1.22 (0.66 – 1.63) | 0.970^#^ |
| **LDH, U/L** | 1204 (937.5 – 1698) | 1141 (899.8 – 1524) | 0.331^#^ | 727.0 (503.8 – 965.0) | 612 (550 – 662) | 0.302^#^ |
| **CRP, mg/L** | 4.45 (2.32 – 6.48) | 3.88 (2.22 – 5.91) | 0.482 | 2.87 (2.87 – 2.87) | 2.42 (1.48 – 7.21) | 1.000^#^ |
| **HbF, %** | 5.40 (3.90 – 7.90) | 8.0 (4.6 – 16.3) | **0.035^#^** | 1.50 (0.97 – 6.05) | 1.20 (0.50 – 1.70) | 0.094 |
| **WBC, /mL** | 11900 (10300 – 12800) | 12300 (9500 – 13975) | 0.938 | 9400 (7550 – 11900) | 9000 (5700 – 10800) | 0.569^#^ |
| **Neutrophil, /mL** | 4736 (3492 – 6500) | 5602 (3190 – 8027) | 0.156 | 3888 (3343 – 5576) | 3600 (2610 – 5500) | 0.622^#^ |
| **Eosinophil, /mL** | 554 (273 – 873) | 436 (208 – 816) | 0.346^#^ | 421 (87 – 965) | 393 (156 – 693) | 0.489 |
| **Lymphocyte, /mL** | 4532 (4096 – 5632) | 4258 (3258 – 5254) | **0.046**^#^ | 3993 (2102 – 6197) | 3366 (2112 – 3828) | 0.245 |
| **Monocyte, /mL** | 819 (553 – 1290) | 1100 (710 – 1452) | 0.185^#^ | 295 (179 – 479) | 768 (500 – 1200) | **0.016** |
| **Platelet, /mL** | 430 (390 – 474) | 414 (337 – 516) | 0.797^#^ | 291 (234 – 386) | 335 (184 – 419) | 0.929 |
| **Total cholesterol, mg/dL** | 123.5 (99.5 – 146.3) | 108.0 (96.25 – 123.0) | 0.056 | 134.0 (92.50 – 156.8) | 128 (99 – 165) | 0.746 |
| **HDL-C, mg/dL** | 34.0 (29.0 – 41.0) | 35.00 (30.25 – 40.50) | 0.593 | 34.00 (29.00 – 45.50) | 38 (34 – 45) | 0.381^#^ |
| **LDL-C, mg/dL** | 68.9 (40.55 (89.15) | 53.20 (43.85 – 70.05) | **0.039** | 61.00 (38.50 – 110.8) | 64.80 (44.60 – 99) | 0.776 |
| **VLDL-C, mg/dL** | 21.0 (15.10 – 27.80) | 18.50 (14.40 – 22.65) | 0.177 | 24.50 (11.50 – 30.75) | 19.40 (14.40 – 25.60) | 0.375 |
| **Triglycerides, mg/dL** | 106 (75.5 – 138.0) | 92.50 (72.0 – 113.3) | 0.183 | 121.5 (57.25 – 153.5) | 97 (72 – 128) | 0.389 |
| **ALT, U/L** | 18.50 (15.25 – 22.75) | 18.50 (15.0 27.25) | **0.010^#^** | 28.0 (28.0 – 28.0) | 12 (9 – 30) | 0.625^#^ |
| **AST, U/L** | 51.50 (41.0 63.75) | 49.0 (35.25 – 69.50) | 0.846 | 33.00 (27.75 – 49.25) | 24 (20 – 29) | 0.436 |
| **ALP, U/L** | 204.5 (107.3 – 345.0) | 135.5 (90.0 174.0) | **0.001** | 407.5 (258.3 – 630.5) | 199 (113 – 284) | **0.011^#^** |
| **NOm, µM** | 26.92 (18.29 – 32.21) | 17.99 (14.96 – 21.66) | **0.003**^#^ | 31.74 (16.53 – 32.29) | 18.15 (13.33 – 19.50) | **0.030** |
| **ET-1, pg/mL** | 4.04 (3.40 – 4.79) | 4.50 (3.19 – 5.96) | 0.249 | 2.39 (2.39 – 2.39) | 5.21 (4.25 – 6.62) | 0.125^#^ |

RBC: Red blood cells; MCV: mean cell volume; MCH: mean corpuscular hemoglobin; MCHC: mean corpuscular hemoglobin concentration; RDW: red cell distribution; LDH: lactate dehydrogenase; CRP: C-reactive protein; HbF: Fetal hemoglobin; WBC: white blood cell; HDL-C: high-density lipoprotein cholesterol; LDL-C: low-density lipoprotein cholesterol; VLDL-C: very low-density lipoprotein cholesterol; ALT: Alanine aminotransferase; AST: Aspartate aminotransferase; ALP: Alkaline phosphatase; NOm: nitric oxide metabolites; ET-1: endothelin-1. SD: Standard deviation. Significant p values are shown in bold (Independent t test). ^#^p value obtained with Mann Whitney *U* test.
