# Supplementary material for: Priapism in sickle cell disease: Associations between NOS3 and EDN1 genetic polymorphisms and laboratory biomarkers
Source: PLoS One. 2021 Feb 4;16(2):e0246067. doi: 10.1371/journal.pone.0246067 (PMC7861393; doi:10.1371/journal.pone.0246067)
Supplement: S3 Table — (DOCX) [file pone.0246067.s003.docx]

**S3 Table.** Laboratory profiles of SCA and HbSC individuals with or without a previous history of priapism (mean and standard deviation).

|  | **SCA individuals (n= 67)** | | | **HbSC individuals (n=21)** | | |
| --- | --- | --- | --- | --- | --- | --- |
|  | **Priapism+**  **(n=31)** | **Priapism-**  **(n=36)** |  | **Priapism+**  **(n=6)** | **Priapism-**  **(n=15)** |  |
|  | **Mean ± SD** | **Mean ± SD** | ***p*** | **Mean ± SD** | **Mean ± SD** | ***p*** |
| **Age, years** | 13.4 ± 5.2 | 14.2 ± 3.8 | - | 9.0 ± 5.7 | 15.2 ± 2.1 | - |
| **RBC, x10^6^/mL** | 2.79 ± 0.49 | 2.69 ± 0.42 | 0.352^#^ | 4.21 ± 0.32 | 4.48 ± 0.50 | 0.253 |
| **Hemoglobin, g/dL** | 8.47 ± 1.33 | 8.41 ± 0.99 | 0.940^#^ | 11.18 ± 1.25 | 12.17 ± 0.96 | 0.066 |
| **Hematocrit, %** | 24.46 ± 4.12 | 25.03 ± 3.37 | 0.532 | 31.55 ± 3.32 | 36.17 ± 3.03 | **0.006** |
| **MCV, fL** | 87.58 ± 9.41 | 93.40 ± 10.97 | **0.024** | 74.88 ± 5.65 | 81.16 ± 5.66 | **0.033** |
| **MCH, pg** | 30.44 ± 3.28 | 31.63 ± 3.74 | 0.175 | 26.50 ± 1.75 | 27.32 ± 2.07 | 0.404 |
| **MCHC, %** | 34.78 ± 1.20 | 33.87 ± 0.90 | **0.000** | 35.42 ± 1.21 | 33.65 ± 0.57 | **0.000** |
| **RDW, %** | 24.44 ± 4.46 | 22.75 ± 3.61 | 0.092 | 19.03 ± 1.11 | 17.91 ± 2.12 | 0.237 |
| **Reticulocyte, %** | 5.85 ± 3.02 | 5.34 ± 2.27 | 0.895^#^ | 3.267 ± 1.36 | 3.34 ± 1.24 | 0.906 |
| **Total bilirubin, mg/dL** | 3.36 ± 1.68 | 3.21 ± 1.19 | 0.726 | 1.432 ± 0.72 | 1.49 ± 0.69 | 0.863 |
| **Direct bilirubin, mg/dL** | 0.46 ± 0.16 | 0.40 ± 0.16 | 0.301 | 0.21 ± 0.09 | 0.30 ± 0.13 | 0.151 |
| **Indirect bilirubin, mg/dL** | 2.89 ± 1.65 | 2.80 ± 1.23 | 0.810 | 1.20 ± 0.67 | 1.33 ± 0.87 | 0.970^#^ |
| **LDH, U/L** | 1306.0 ± 468.1 | 1191.0 ± 383.5 | 0.331^#^ | 740.8 ± 289.2 | 625.8 ± 115.2 | 0.302^#^ |
| **CRP, mg/L** | 4.18 ± 3.37 | 4.72 ± 3.15 | 0.482 | 4.02 ± 2.13 | 4.26 ± 3.99 | 1.000^#^ |
| **HbF, %** | 6.7 ± 5.7 | 9.6 ± 6.1 | **0.035^#^** | 3.3 ± 3.8 | 1.4 ± 1.1 | 0.094 |
| **WBC, /mL** | 11841 ± 2038 | 11895 ± 3419 | 0.938 | 9433 ± 2585 | 8993 ± 4305 | 0.569^#^ |
| **Neutrophil, /mL** | 4972 ± 1976 | 5849 ± 2866 | 0.156 | 4241 ± 1157 | 4429 ± 2782 | 0.622^#^ |
| **Eosinophil, /mL** | 774 ± 963 | 582 ± 508 | 0.346^#^ | 601 ± 715 | 436 ± 370 | 0.489 |
| **Lymphocyte, /mL** | 5054 ± 1636 | 4235 ± 1412 | **0.046**^#^ | 4157 ± 2103 | 3252 ± 1317 | 0.245 |
| **Monocyte, /mL** | 915 ± 510 | 1117 ± 579 | 0.185^#^ | 342 ± 244 | 816 ± 407 | **0.016** |
| **Platelet, /mL** | 436 ± 108 | 424 ± 113 | 0.797^#^ | 318 ± 142 | 324 ± 134 | 0.929 |
| **Total cholesterol, mg/dL** | 123.4 ± 30.14 | 111.1 ± 23.28 | 0.056 | 127.2 ± 37.24 | 132.9 ± 35.51 | 0.746 |
| **HDL-C, mg/dL** | 34.23 ± 8.92 | 35.31 ± 7.30 | 0.593 | 36.33 ± 7.84 | 41.00 ± 14.44 | 0.381^#^ |
| **LDL-C, mg/dL** | 69.80 ± 30.24 | 56.82 ± 19.72 | **0.039** | 68.17 ± 37.01 | 72.33 ± 27.09 | 0.776 |
| **VLDL-C, mg/dL** | 21.31 ± 7.60 | 18.99 ± 6.11 | 0.177 | 22.67 ± 10.11 | 19.53 ± 5.73 | 0.375 |
| **Triglycerides, mg/dL** | 106.40 ± 37.94 | 94.94 ± 30.55 | 0.183 | 113.0 ± 51.26 | 97.67 ± 28.67 | 0.389 |
| **ALT, U/L** | 20.80 ± 9.48 | 20.85 ± 9.26 | **0.010^#^** | 28.00 ± 0.0* | 21.40 ± 24.44 | 0.625^#^ |
| **AST, U/L** | 52.70 ± 14.87 | 51.89 ± 18.94 | 0.846 | 36.67 ± 10.56 | 30.80 ± 23.16 | **0.021^#^** |
| **ALP, U/L** | 248.0 ± 184.5 | 135.8 ± 58.17 | **0.001** | 467.0 ± 287.3 | 209.7 ± 101.4 | **0.011^#^** |
| **NOm, µM** | 27.94 ± 13.97 | 19.16 ± 6.73 | **0.003**^#^ | 25.88 ± 9.46 | 17.41 ± 6.06 | **0.030** |
| **ET-1, pg/mL** | 4.15 ± 1.01 | 4.66 ± 1.74 | 0.249 | 4.71 ± 2.48 | 5.63 ± 2.04 | 0.125^#^ |

RBC: Red blood cells; MCV: mean cell volume; MCH: mean corpuscular hemoglobin; MCHC: mean corpuscular hemoglobin concentration; RDW: red cell distribution; LDH: lactate dehydrogenase; CRP: C-reactive protein; HbF: Fetal hemoglobin; WBC: white blood cell; HDL-C: high-density lipoprotein cholesterol; LDL-C: low-density lipoprotein cholesterol; VLDL-C: very low-density lipoprotein cholesterol; ALT: Alanine aminotransferase; AST: Aspartate aminotransferase; ALP: Alkaline phosphatase; NOm: nitric oxide metabolites; ET-1: endothelin-1. SD: Standard variation. *Due to sample limitation, only one patient is showed. Significant p values are shown in bold. p value obtained with Independent t test or ^#^p value obtained with Mann Whitney *U* test.
